# High-Resolution Pulse Oximetry and Titration of a Mandibular Advancement Device for Obstructive Sleep Apnea

**DOI:** 10.3389/fneur.2019.00757

**Published:** 2019-07-17

**Authors:** James E. Metz, Hrayr P. Attarian, Mickey C. Harrison, James E. Blank, Christopher M. Takacs, Dale L. Smith, David Gozal

**Affiliations:** ^1^The Metz Center for Sleep Apnea, Columbus, OH, United States; ^2^Circadian Rhythms and Sleep Research Lab, Northwestern University Feinberg School of Medicine, Chicago, IL, United States; ^3^Department of Behavioral Sciences, Olivet Nazarene University, Bourbonnais, IL, United States; ^4^Department of Child Health, University of Missouri School of Medicine, Columbia, MO, United States

**Keywords:** intraoral appliances, mandibular advancement device, obesity, outcomes, sleep apnea, high-resolution pulse oximetry, titration

## Abstract

**Background:** To determine whether utilizing high-resolution pulse oximetry is a viable method for evaluating the successful titration of oral appliances for the treatment of obstructive sleep apnea (OSA) patients.

**Methods:** Of 136 consecutive potentially eligible OSA patients, 133 were fitted with mandibular advancement devices (MADs), and 101 completed all phases of treatment. The vertical and horizontal dimensions of the appliances were adjusted based on three-nights with a high-resolution pulse oximeter during sleep and associated software after each adjustment.

**Results:** Significant improvements in OSA severity were apparent in patients at all disease severity levels. High-resolution pulse oximetry provided reliable guidance in the titration process of mandibular advancement therapy. In 67 subjects (66.3%), a respiratory event index of <5 events/hour was achieved.

**Conclusions:** OSA can be effectively treated with a MAD at any severity level, and high-resolution pulse oximetry provides critical information to guide oral appliance titration.

## Introduction

Obstructive sleep apnea (OSA) is a prevalent and relatively underdiagnosed condition. According to the criteria used, OSA may affect 12–50% of adults and is characterized by recurrent episodes of partial and complete airway obstruction during sleep (1–3). As of 2012, 82% of men and 93% of women with OSA were estimated to be undiagnosed (4). The apnea-hypopnea index (AHI), as derived from an overnight polysomnogram (PSG), is the most commonly used measure of disease severity, with an AHI <5 events/hour considered normal, 5–14.9 events/hour considered mild, 15–29.9 events/hour considered moderate, and ≥30 events/hour defined as severe OSA (5). However, AHI may not necessarily capture pathophysiologic information on why upper airway obstructions occur in any given patient (6). Patients with OSA are subjected to intermittent hypoxia, sympathetic activation, and sleep fragmentation, which if left untreated, are independently associated with cardiometabolic disturbances, neurocognitive deficits, motor-vehicle, and work-related accidents, insomnia, anxiety and depression, and ultimately, increases in the risk of pre-mature death (5, 7–11).

Historically, mandibular advancement devices (MADs) have been employed to treat snoring and mild to moderate OSA. More recent evidence points to MADs being a viable option even for patients with severe OSA, especially in cases of intolerance or poor continuous positive airway pressure (CPAP) therapy adherence (12–15). Treatment outcomes of MADs vary greatly, and there is no consensus as to an ideal method of optimization. Myriad titration options exist (16), including home advancement followed by further titration during a PSG (17, 18); remote-controlled mandibular protrusion during a PSG (19, 20); endoscopy to evaluate changes in airway anatomy with a MAD in place (21); titration of a temporary appliance, with either one or multiple nights of PSG study (22, 23); and a home-based study evaluating multiple respiratory channels (24–26). Successful MAD titration was also reported based on a combination of the resolution of subjective patient-reported symptoms along with stand-alone pulse oximetry (27). Here, we developed and implemented a novel, low-cost MAD titration approach using a stand-alone high-resolution pulse oximeter (HRPO) via interpretive software to adjust the settings of the MAD and thus optimized the treatment of otherwise CPAP non-adherent or intolerant OSA at all severity levels. The goal of this study is to detail a procedural approach that illustrates how a MAD can be used as a true medical device; and titrated to a high degree of accuracy, as defined by the American Academy of Sleep Medicine (AASM) for CPAP success, which has not previously been shown to our knowledge.

## Materials and Methods

### Sample

One hundred and thirty-six consecutive subjects were prospectively evaluated for this case series study. Three subjects were excluded because their pre-treatment respiratory event index (REI) was <5 events per hour. A total of 133 patients with OSA who had either failed or refused CPAP therapy were evaluated in a private practice general dentistry office in Columbus, Ohio, USA, over a 35-month span between January 2013 and November 2015. All patients had received a medical diagnosis of OSA from a board-certified sleep physician, and each patient had a letter of medical necessity/prescription for oral appliance therapy from their physician. One hundred and one subjects completed all phases of the study protocol, including post-titration efficacy sleep testing. The study was carried out in accordance with recommendations of the Institutional Review Board, Feinberg School of Medicine: Northwestern University (Chicago, IL, USA). The protocol was approved by Panel C of the Institutional Review Board at Northwestern. All subjects gave written informed consent in accordance with the Declaration of Helsinki prior to initiating treatment. OSA was clinically diagnosed either via an overnight polysomnogram (PSG) in an AASM-accredited sleep laboratory or through home sleep testing (HST) (SleepView, Type III, eight-channel monitor, CleveMed: Cleveland Medical Devices, Inc., Cleveland, OH, USA), pre- and post-treatment respiratory indices were blindly converted to the REI following AASM standards, and interpreted by a board-certified sleep medicine specialist who was blinded to all components of the study.

### Treatment and Titration Protocol

**Figure 1:**
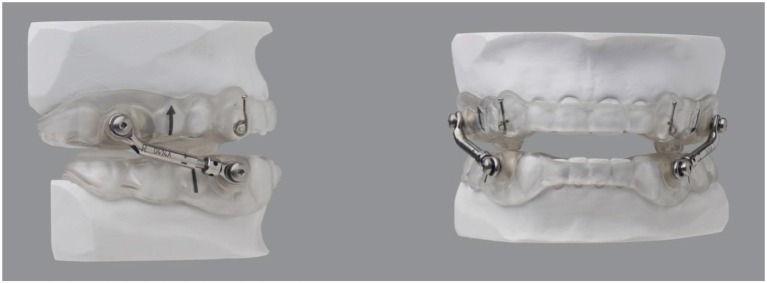
Herbst-style MAD used for all patients.

Intake examinations included medical, dental and sleep histories, as well as the Epworth Sleepiness Scale (ESS) to assess excessive daytime sleepiness (EDS). Maxillary and mandibular impressions were acquired, along with a forward (protrusive) bite registration at ~3 mm. A MAD was fabricated for each patient (Figure 1). At insertion, each tray was verified for fit and comfort, and Herbst-style attachments were utilized to join the trays. The MAD of “Herbst” design was made consistently, customized, and minimal in design with all excess plastic and metal removed, which did not impact durability.

**Figure 2:**
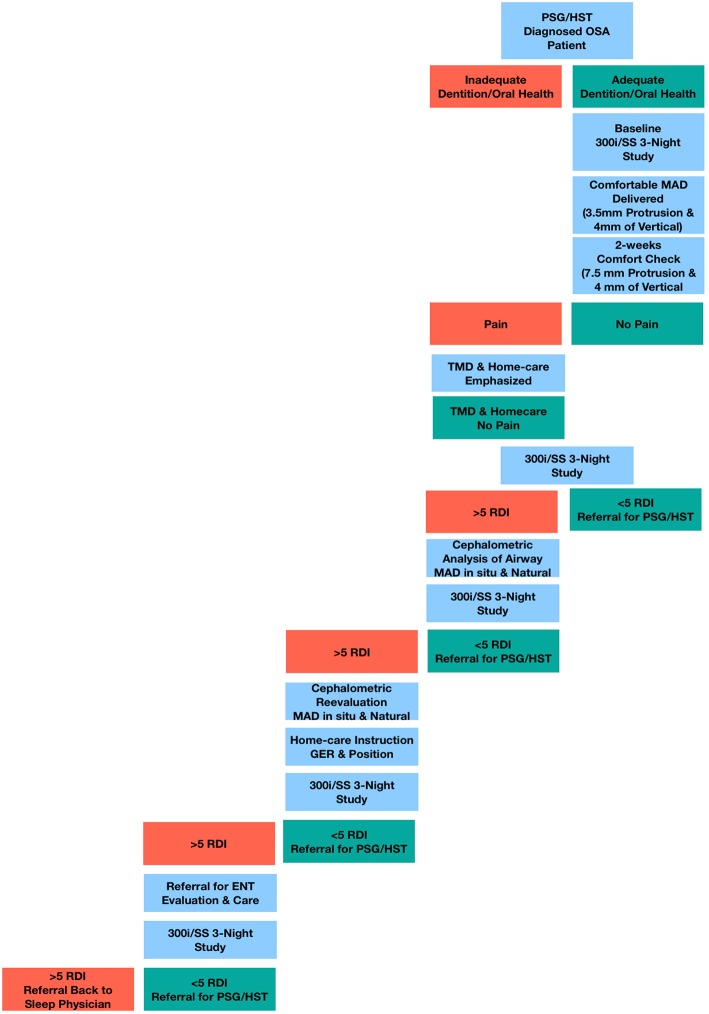
Titration protocol flow chart.

Initial protrusion was set at 3.5 mm from where the teeth best fit together (habitual occlusal) (Figure 2). Three and one-half millimeters was selected as the starting point to ensure tolerability of the device and to limit the development of side effects. The horizontal protrusion was increased during the titration process over the subsequent weeks. The starting vertical dimension was set at 4 mm, measured from the buccal cusp points of the maxillary and mandibular first premolars, and included the thickness of each tray (1 mm each) plus a 2 mm acrylic pad. Elastics were initially employed to help control mouth gaping, enable lip sealing and promote nasal breathing during sleep. One of the identified indications of possible treatment success was when the elastics were no longer required to keep the jaw closed, a feature that has been observed by other clinicians (28).

**Figure 3:**
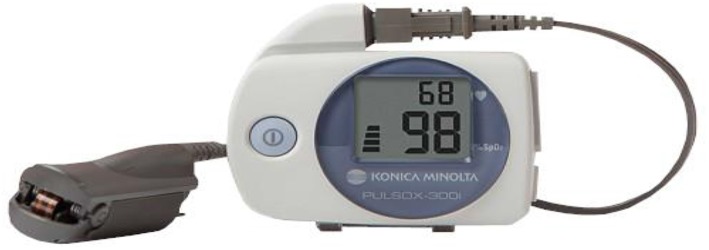
Minolta 300i high-resolution pulse oximeter with finger probe.

Patients were appointed every 2–4 weeks for adjustment and to assess side effects of MAD therapy and subjective symptom relief; especially those related to temporomandibular (TM) joint pain/temporomandibular disorder (TMD). There appeared to be a link between absence of jaw pain and a marked reduction in the heart rate as reported on the SatScreen studies. After 6–8 weeks and once patients reached the identified horizontal treatment range of 5–7 mm, SatScreen software (Patient Safety, Inc., Columbus, OH, USA) was employed with a Minolta 300i HRPO equipped with the SR-5C finger clip probe (Konica Minolta Sensing, Inc., Osaka, Japan) for three nights of a Minolta 300i/SatScreen (300i/SS) study with the MAD in place (Figure 3). The 300i and SR-5C can store up to 300 h of data, peripheral capillary oxygen saturation (SpO2) memory resolution is 0.1%, 1 s interval measurements are recorded, and are user friendly, not requiring a high level of skill to operate.

**Figure 4:**
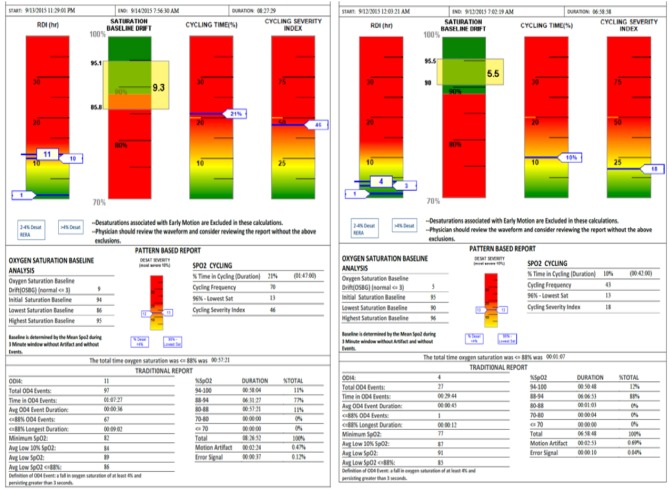
SatScreen report (Left RDI 13—Prone Sleeper, Right RDI 4—Left Side Sleeper) shows the difference between two body positions with the appliance adjustment unchanged.

SatScreen reports provide estimates of the respiratory disturbance index (RDI); SpO2 recorded to 0.1% values including minimum, maximum and fluctuations, based on an oxygen desaturation of 2–4%; pulse rate; cycling time and cycling severity; and motion artifact detection via actigraphy and the SatScreen algorithm, which identifies and discards physiologically impossible recordings due to movement (Figure 4). The SatScreen algorithm and the 300i's 0.1 s data collection speed permits near elimination of movement error, which is not possible with standard oximetry recordings. The algorithm provides interpretation of the slope of the SpO2 waveform, giving a stable monitoring method for titration. It was noted that movement reduced as the patient's OSA severity improved, which was a helpful indicator of treatment outcomes.

The averaged data from the three-night recordings were evaluated to guide the decision as to whether further titration and additional 300i/SS studies were warranted. The reason for three nights of 300i/SS study included the ability to exclude an anomalous night due to loss of power (battery failure); detachment of the finger probe; factors such as alcohol/substance use or abuse; medications; environmental allergies; or any other potential documented disturbances in the bedroom or sleep during any of the three nights. All technically acceptable data were then averaged and summarized. Subjects kept sleep diaries during the oximetry testing, to match objectively noted anomalous readings on the SS reports with subjectively logged contributing factors, such as illness, medication, or substance ingestion.

Polysomnogram is the gold standard to assess sleep physiological parameters but was born in the laboratory. It is difficult, if not impossible, to use for a clinical/home based application, and the issue of cost and insurance coverage is of obvious importance as well. The decision to utilize the Minolta 300i and SatScreen could be implemented at any point according to the progress of an individual's MAD titration. The 300i eliminated the need to schedule an appointment with the sleep laboratory, and alleviated the concern for cost or insurance reimbursement. The loss rate of data from the three-night oximetry protocol averaged 37%. If two nights with an error detection rate <10% were not achieved, testing was repeated. SatScreen calculates the amount of time lost due to patient movement or probe malfunction and recommends that the study be repeated with >10% error.

As the patient approached ideal treatment position (that is, horizontal protrusion of 5–7 mm and vertical dimension of 4 mm) for the MAD, desaturations became much more subtle (29). SatScreen measures respiratory effort-related arousals (RERAs) in the 2–4% range to record milder events, which would be detected on a polysomnogram, but missed on other oximetry reporting systems. The ability to detect minor desaturations in the 2–4% range is also important for screening, to allow for milder cases to be recognized; and in titration, as events move from more severe to less severe with successful application of the oral appliance, it is crucial to have these 2–4% events resolved by MAD to decrease autonomic arousals and the associated elevations in heart rate (30, 31) SatScreen keeps the 2–4 and >4% desaturations categorized for clarity. Tracking these more minor events proved to be beneficial. If the individual's parameters, no additional titrations were needed after the final oximetry night of titration with 300i/SS. A clearing study was performed with PSG/HST as per clinical protocol and in order to further confirm with standard tests based on guidelines, but did not change the titrated MAD position from the 300i/SS.

**Figure 5:**
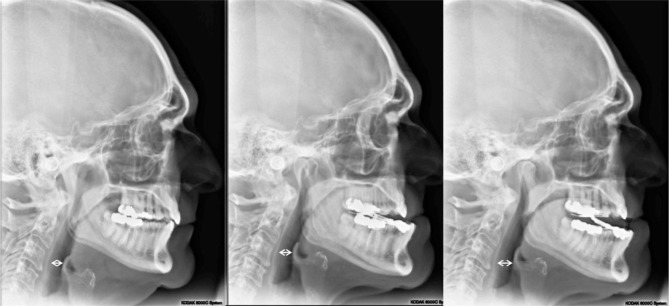
Lateral cephalometric radiographs: **(Left)** Pretreatment image; **(Middle)** minimum vertical dimension of the oral appliance (2 mm), 9 mm PAS; **(Right)** vertical dimension of +2 mm added to the oral appliance (4 mm total), 12 mm PAS. PAS is defined as the shortest horizontal distance between the posterior pharyngeal wall and the base of the tongue (33).

Titration of the MAD has only two data points for titration that were discoverable in this study. First, the 300i/SS sleep study reports and second the digital “One Shot” lateral cephalometric radiographic imaging capturing the entire oropharyngeal area in a single pulse of radiation. The cephalometric radiograph was employed as an adjunct to 300i/SS during the MAD adjustment process. One of the authors (JM) developed a protocol for the reproducibility of the cephalometric image. In this protocol, the patient stands perfectly straight with ear posts of the cephalometric unit properly inserted, teeth closed together maximally; inhales, then exhales, taking care not to forcibly exhale past the end-expiratory point; does not swallow; and maintains an ideal head posture by looking straight ahead into his/her own pupils in a mirror mounted at eye level. Only then is the radiograph exposed. These radiographs are taken at pre-treatment, as well as during the MAD titration sequence with the device *in situ*, to assess the impact of various horizontal and vertical dimensions of the appliance on the posterior airway space (PAS), which is defined as the shortest horizontal distance between the posterior pharyngeal wall and the base of the tongue or epiglottis [(32); Figure 5]. In general, three cephalometric images were acquired during the process to determine maximum pharyngeal opening as corroborated by 300i/SS recording, with these two metrics being required to agree as criterion for MAD successful titration.

The cephalometric radiograph process is not designed to diagnose deficient airways from one subject to another. During the titration process, the 300i/SS served as the guide to titration. If the reports showed a problem with events, the cephalometric radiograph was utilized to assist with estimates of airway space. It lends insight as to the impact of the MAD at various adjustments on the physical dimensions of the PAS from initial findings within the treatment sequence for one individual. Strict adherence to the exposure protocol appears to eliminate errors associated with capturing the PAS for that adjustment. The end-expiratory point of the breath is the maximum area of the PAS (33). A cone beam computed tomography (CBCT) was available for use, but no correlation could be gained between CBCT image and treatment position, which was possible only with the lateral cephalometric radiograph with a full image receptor. The cephalometric partial or bar image receptor gave inconsistent radiographic images as the scan required 7 s. On average the radiation exposure of the patient was also less with the lateral cephalometric radiographs than with CBCT.

As mentioned, incorporating both the 300i/SS data and the lateral cephalometric radiograph was critical to the successful titration of the MAD with the protocol presented. The oximetry data with SatScreen allowed for the consideration of many common parameters including: oxygen desaturation index (ODI4), desaturations below 90% SpO_2_; and the most useful feature of the SatScreen report, the indication of physiologic parameters in the green portion of the graphs. If the resultant numbers were “in the green” it was a reliable indication the patient was properly titrated. The SatScreen reports respiratory disturbance index (RDI); the baseline drift of the SpO_2_; SpO_2_ cycling time, where one desaturation event occurs within 10 s of another; and severity index, in which the patient does not return to the baseline level of SpO_2_. Each of the four previously mentioned parameters is represented in “stoplight” form. The red color is severe, yellow is caution, and the green is within the range of normal. The clinician is therefore provided a quick assessment of the patient's condition at a glance with this colored scoring.

When the SatScreen report was within normal ranges and the patient asymptomatic, a referral was made for the clearing PSG or HST study with the sleep physician. Temporomandibular (TM) joint pain/TMD symptoms appeared to be related to an elevated pulse rate (pulse cycling), which is also reported in graph form on the SatScreen report (27). The highly active heart occurred in >90% of the patients with complaint of TM joint pain/TMD. If TM joint pain or other symptoms were present, titration continued.

Following the resolution of objective symptoms as evaluated by 300i/SS and subjective symptoms as measured by the ESS and self-reported TM joint pain, the patient was referred for a follow-up PSG or HST. The ODI4 many times tended to nearly duplicate the PSG report at initiation of treatment but the heart rate and “stoplight” concurred with the patient's symptoms at the end of treatment. A benchmark for referral for a clearing study was at least an 80% reduction of the RDI from the 300i/SS report. Titration time varied with each patient and their compliance with treatment. Compliance included adherence to home care instructions, keeping scheduled appointments, and 300i/SS use. Consequently, the time of titration varied; the range of time for referral for clearing study was 6 weeks to 9 months with an average of 10 weeks. Despite improvement of SatScreen parameters, some patients were still symptomatic, and required longer duration of interventions or additional interventions for their resolution.

Polysomnogram (PSG) is considered the gold standard for diagnosis and efficacy testing of OSA (34), but the home sleep testing via portable unattended cardiorespiratory monitors may provide accurate diagnosis and efficacy data and is increasingly utilized (34, 35). HST offers the benefits of cost-efficient testing in the home sleep environment (36, 37) as well as being recognized as non-inferior to PSG (38, 39). For analysis purposes and continuity of the sleep data collected all pre- and post-treatment respiratory indices were blindly converted to the REI, based on total monitoring time (MT) [(number of respiratory events)/MT in hours] (40). Rescoring the data enabled the PSG results to be rescored as HST data.

Once the efficacy of the MAD was confirmed, individuals were placed on a recall program to regularly assess continued treatment success with the device and to reinforce sleep hygiene, sleep position and weight control as part of their overall management (41). While positional OSA was not a reported variable of this study, left lateral sleep position, preferably on an incline (MedCline, 8825 Rehco Road Suite D, San Diego, CA 92121) (42, 43) was promoted with every patient undergoing MAD therapy, to attempt to alleviate supine sleep and reduce GER. As patients were sleeping in their home environments, it was not possible to objectively determine whether they were compliant with sleep position recommendations.

### Statistical Analysis

Statistical analyses were conducted in Stata software (version 14) (Statacorp LLC, College Station, TX). All data were scored and analyzed in a blinded fashion. The data were analyzed using means and frequencies to examine sample characteristics. Then, frequencies were used to determine the percentage of MAD complete responders (complete response [CR]-[REI] < 5), partial responders (partial response [PR]-REI ≥ 50% reduction), and non-responders (non-response [NR]-REI < 50% reduction or increase). *T*-tests for independent means and chi-square tests for proportions were used to examine differences in demographics and disease characteristics [REI, body mass index (BMI)] among subjects with CR, PR, and NR. Logistic regression models were also constructed to evaluate the independent effects of age, sex, obesity, amount of protrusion, and vertical component of mandibular advancement on the odds of MAD CR, PR or NR. Pre-post-differences, as well as differences in trends across individuals based on demographic variables, were examined through repeated measures ANCOVA. Statistical significance was defined as *p* < 0.05 on two-tailed tests.

The PSG/HST recordings were closely related to those of the Minolta 300i/SatScreen, and the results would typically be similar between the two measures (*r* = 0.578, *p* < 0.001).

## Results

A total of 101 OSA patients completed the MAD treatment protocol (Table 1). Of these patients, 77 (76.2%) had tried CPAP but could not tolerate the therapy; 20 (19.8%) had refused CPAP without attempting it; and 4 (4%) were currently using CPAP successfully but requested a MAD as an alternative treatment to use at their discretion and utilized MAD exclusively during all studies. An additional group of 32 patients underwent the MAD titration protocol but was either unable or unwilling to undergo final efficacy testing with a PSG or HST.

**Table 1:** Demographic and clinical characteristics of 101 subjects completing the MAD titration protocol.

	**Total cohort**	**Subjects with CR (<5/h)**	***P*-value**	Subjects with PR or NR^*^
Mean baseline REI (events/hour)	28.13 ± 23.73	21.43 ± 16.75	<0.001	41.32 ± 29.58
Mild OSA	43 (42.6%)	34 (81%)	<0.001	9 (19%)
Moderate OSA	20 (19.8%)	18 (90%)	0.005	2 (10%)
Severe OSA	38 (37.6%)	18 (47.4%)	0.001	20 (52.6%)
Total (*n*)	101	67 (66.3%)		34 (33.7%)
Mean age (years)	55.14 ± 11.14	53.12 ± 10.99	0.010	59.12 ± 10.48
Gender (F)	42 (41.6%)	33 (50.8%)	0.006	9 (25%)
BMI (kg/m^2^)	30.48 ± 6.46	29.81 ± 6.29	0.150	31.77 ± 6.66
Normal weight	13 (12.9%)	11 (16.9%)		2 (5.6%)
Overweight	42 (41.6%)	27 (41.5%)		15 (41.6%)
Obese	37 (36.6%)	23 (35.4%)		14 (38.9%)
Severely obese	9 (8.9%)	4 (6.2%)		5 (13.9%)
Horizontal adjustment (mm)	6.45 ± 1.60	6.10 ± 1.44	0.002	7.13 ± 1.69
Range	2–11	2–11		4.8–11
Vertical adjustment (mm)	3.98 ± 0.93	3.88 ± 0.90	0.150	4.16 ± 0.95
Range	2–6	2–6 mm		2.5–6

*Sixty-seven subjects achieved CR, and there were 34 subjects were PR or NR. Percentages for Complete Responders (CRs) are their proportions within the overall cohort*.

* *Partial Responders and Non-Responders (PNRs) are their proportions within each category of severity not in the overall cohort*.

Pre-MAD, the mean REI was 27.6 events/hour (SD = 8.44), and the median REI was 17.3 events/hour (range: 5.0–90.3 events/hour). Forty-two (41.6%) subjects had mild OSA with an REI between 5 and 14.9 events/hour; 21 (20.8%) had moderate OSA with an REI between 15 and 29.9 events/hour; the remaining 38 patients (37.6%) had severe OSA with REI ≥ 30 events/hour. Women comprised 41.6% (*n* = 42) of the cohort. The mean age was 55.1 years old (SD = 11.08), and the median was 57 years. The mean BMI was 20.6 kg/m^2^ (SD = 6.47), and the median was 29.2 kg/m^2^. Of the entire cohort, 12 patients (12.9%) were of normal weight with a BMI between 19.3 and 24.2; 42 patients (41.6%) were overweight with a BMI between 25 and 29.8 kg/m^2^; 37 patients (36.6%) were obese (BMI 30–39.9), and nine patients (8.9%) were severely obese with BMI ≥ 40 kg/m^2^.

**Figure 6:**
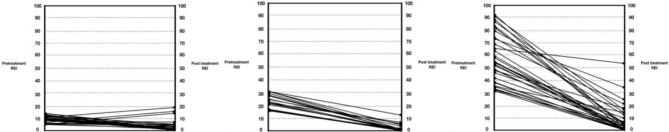
Pre-treatment and post-treatment respiratory event index (REI) values for the participants in the study. The left graph is mild (REI < 15); middle is moderate (REI 15–30); and right is severe (REI > 30).

For the cohort, the overall reduction in the REI after MAD use was significant (difference 95%CI: 18.86–27.65; *p* < 0.001). Time-by-demographic variable interactions indicated that the trends differed according to age (*p* = 0.02) and sex (*p* = 0.01), but not BMI (*p* = 0.50). Older patients and men demonstrated greater pre-post-MAD reductions in REI, likely due to higher initial values. The primary outcome was CR, as defined by a post-treatment REI of <5 events/hour. Of the 101 patients, 67 (66.3%) fulfilled the CR criteria; 24 patients (23.8%) exhibited PR and had their REI drop by >50% but remain above five events/hour; 10 patients (9.9%) had their REI not change, decrease by <50% or, in rare cases, increase (NR). Almost half of the patients with CR (32 subjects or 49.2%) had mild OSA at baseline; 23.1% (*n* = 16) had moderate OSA at baseline; and 17 (29.2%) had severe OSA at baseline. The mean REI of the CR subjects was 21.8 events/hour (SD = 17.05), and the median was 15.1. Eighty-six of 101 subjects (85.1%) attained an REI of <10 events/hour, and 86/101 achieved a ≥50% reduction in REI (Table 1). Figure 3 illustrates response based on initial disease severity of mild, moderate, or severe (Figure 6).

The average number of nights of 300i/SS assessments during appliance titration was 5.7 (range: 2–11), with some nights of the 300i/SS study being excluded due to loss of contact with the finger probe or loss of battery power. The average final value of the respiratory disturbance index (RDI-apnea+hypopnea+respiratory effort-related arousals) for the 300i/SS studies prior to efficacy studies for the complete responders was 5.89 events/hour (range: 0.5–23.3). Correlations between post-300i/SS and post-ESS scores were not significant for the full sample (*r* = 0.12, *p* = 0.24) or among complete responders (*r* = 0.11, *p* = 0.38). The ESS scores for CR were 7.30 (average pre-treatment; range: 0–18) and 4.88 (average post-treatment; range: 0–19), demonstrating an overall improvement in subjective symptoms (*p* < 0.001). Three lateral cephalometric radiographs (for the baseline, initial MAD placement, and final titration) were typically acquired.

The mean age of complete responders was 53.1 years (SD = 10.99), and the median was 57 years. Responders were significantly younger than non-responders (*M* = 59.12, SD = 10.48, 95%CI: 1.48–10.52, *p* = 0.01). The CR male/female ratio was ~1/1 (35 men and 32 women), although participating women had a higher percentage of subjects with CR or PR (78.05%) than men (58.33%, p = 0.039). The responders' mean BMI was 29.89 kg/m^2^ (SD = 6.243), and the median was 28.8 kg/m^2^. No significant differences existed in BMI between responders and non-responders (95%CI: −4.65 to 0.72, *p* = 0.16).

**Figure 7:**
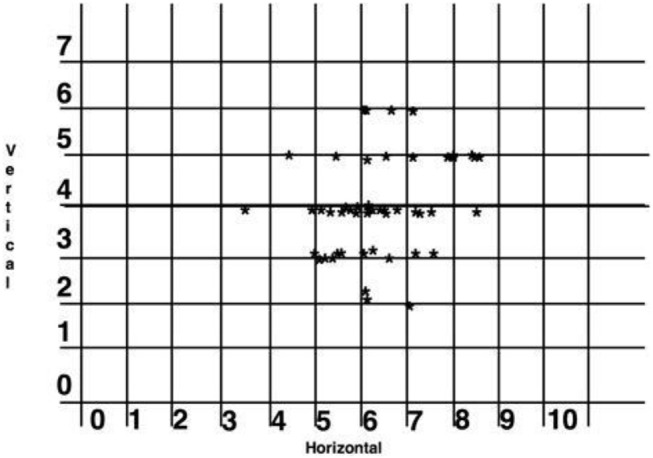
Scatter graph of the horizontal protrusion and vertical dimension of complete responders (in millimeters). One subject was excluded at 11 mm horizontal and 4 mm vertical as lying outside the range.

Based on the titration data from the 300i/SS studies, lateral cephalometric radiographs, and ESS scores, successful horizontal protrusion was found to be an average of 6.10 mm, and the optimal vertical dimension was considered to be 4 mm. The majority of the patients fell within the therapeutic range of these dimensions. In addition, responders had a smaller MAD protrusion (*M* = 6.10 mm, SD = 1.44) than non-responders had (*M* = 7.13 mm, SD = 1.69, 95%CI: 0.38–1.66, *p* = 0.002), although no differences emerged in the vertical component of the MAD devices (95%CI: −0.66 to 0.10, *p* = 0.17) (Figure 7).

**Figure 8:**
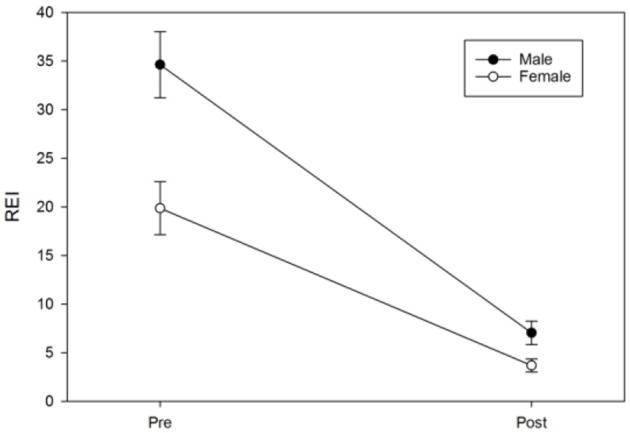
Sex differences in pre-post REI trends. The error bar represents the standard error.

**Figure 9:**
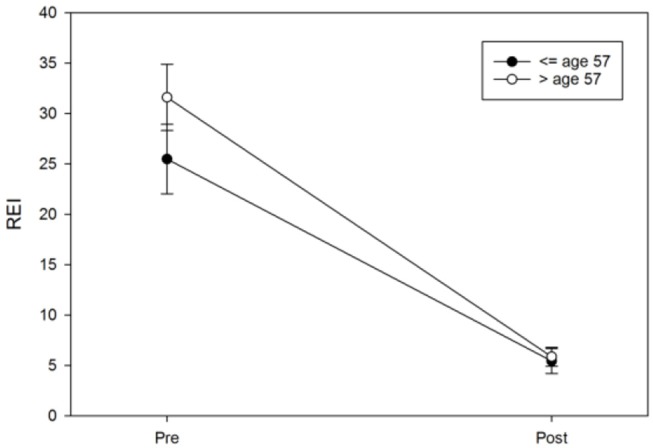
Age differences in pre-post REI trends. An age median split at 57 years was used only for illustrative purposes in this figure; the error bars represent the standard error.

Logistic regression was next performed and included age, protrusion of the mandibular device, sex, the vertical component, and BMI to examine each predictor while adjusting for the other variables in a model predicting the MAD response. The results suggested that only age (OR = 0.94, 95%CI: 0.89–0.99, *p* = 0.017) and protrusion of a MAD (OR = 0.67, 95%CI: 0.49–0.92, *p* = 0.012) were significant predictors of the MAD response (Figures 8, 9). Neither sex (OR = 95%CI: 0.84–7.85, *p* = 0.09) nor BMI (OR = 95%CI: 0.86–1.01, *p* = 0.08) were significant predictors, suggesting that these commonly cited predictors may not be valuable when adjusting for other variables. The vertical component of the device did not vary significantly from the 4 mm range (OR = 95%CI: 0.63–2.10, *p* = 0.63) Additionally, although the above pre-post analyses indicated sex differences in REI trends, the post-treatment REI values were quite similar (see Figure 8), which may be reflected in the lack of sex differences in the odds of MAD response when adjusting for other controllable variables.

Notably, 32 of the non-responders were in fact classified as NR because of their reluctance to undergo HST or a PSG at the end of their MAD titration. However, their 300i/SS findings at the end of titration were similar to those of the responders (*p* > 0.05).

## Discussion

Oral appliance therapy for OSA with a MAD is an important treatment option for patients who are either intolerant to or reject CPAP. Studies have shown that nearly half of patients prescribed CPAP are non-adherent at 1 year of use, and 15 to 30% of those diagnosed with OSA reject CPAP without an attempt (44). The high success rate with TM joint pain/TMD patients may offer an effective method for the treatment of this condition as well. AHI uses a 4% desaturation as one of the parameters to define the severity of OSA, and as the obstruction resolves with the MAD, the desaturations become less severe and frequent with many of these events becoming <4%. Therefore, the use of ODI four was deemed as inappropriate for refining the titration procedures, with changes in heart rate displaying evident utility at the end of titration and being more closely associated with symptomatic relief, improvements in residual daytime sleepiness and TM joint pain/TMD (45).

The primary outcome implemented in the present study was a post-treatment reduction of the REI to <5 events/hour, which was achieved in 66.3% of the participants, with no significant differences detected across OSA severity categories. A larger proportion of the cohort (85.1%) achieved a post-treatment REI of <10 events/hour. Here, the magnitude of horizontal protrusion, but not the vertical dimension, was found to be significantly associated with CR. These results suggest that the vertical height of 4 mm falls into an anatomic norm and that changing this measurement would, quite likely, lead to a lesser outcome. Importantly, BMI did not emerge as a significant factor for MAD complete response; this finding is in stark contrast to the results of previous studies suggesting that a higher BMI contributes to reduced success with oral appliance therapy for OSA (46–48). The potential reasons for the discrepant findings are unclear, since the cohort consisted of consecutive patients and was not specifically selected for any of the *a priori* predictors of unfavorable responses to MADs.

Many methods can be employed to adjust a MAD for the treatment of OSA. This study utilized a novel method of titration combining lateral cephalometric images, along with stand-alone Minolta 300i/SatScreen. Benefits of using 300i/SS for appliance adjustment include the following: multiple nights of an 300i/SS study can be performed to account for inconsistent events and night-to-night variability by taking an average of three nights; the device is comfortable; patients are studied in their home sleep environment, diminishing the first-night effect (37, 49); there is minimal expense to the patient and medical provider; and if results are not optimal, further adjustment to the device can be made and 300i/SS dispensed again, to demonstrate efficacy prior to the final clearing PSG/HST. The 300i/SS findings served as a significant predictor of MAD success. Indeed, 300i/SS results in the last oximetry assessment predicted the responder status (if not controlling for any other variables, *p* = 0.001; if adjusting for age, protrusion, gender, vertical, and BMI, *p* = 0.029). The ESS was used to demonstrate improvements in subjective symptoms. Lateral cephalometric radiographs, following a strict exposure protocol, were used to visualize the impact of the MAD on the PAS. Utilization of the lateral cephalometric radiograph allowed for objective assessments of the increase of the space between the hard/soft palate and the tongue along with the changes in PAS.

The number of iterative overnight oximetry assessments and MAD adjustment sessions between the initial evaluation and the final PSG or HST could have been much higher. However, this was not the case, whereby the mean number of intermediary sessions and total duration of MAD titrations in CR, PR, and NR were remarkably similar, indicating that the comparatively favorable CR rates in this cohort were not the result of more prolonged and labor-intensive efforts, but rather reflect individual factors across CR and non-CR, namely, age and the degree of horizontal protrusion achieved. Thus, utilizing 300i/SS to titrate MADs may facilitate more objective adjustments to enhance and streamline the MAD titration, ultimately resulting in more favorable CR rates.

Recognizing that Fleury et al. (27) previously published on pulse oximetry and oral appliance titration, there are important differences between their process and our current technique. Fleury et al. used AHI <10 events/hour as criteria for successful titration; they advanced by 1 mm every week until resolution of symptoms and reduction in ODI3% to <10 events/hour, or until maximum comfortable protrusion was obtained. Our protocol used ODI4% in combination with the SatScreen algorithm, as required by AASM and Medicare, and AHI <5/hour, which sets a much higher bar for success. Fleury et al. reported no measurement of the vertical dimension of the appliance, which we demonstrated to be critical for a successful outcome with this device and technique.

It is noteworthy that the majority of the patients fell into 4 mm of vertical dimension with the utilization of the MAD described. A few people were as much as 6 mm. Small statured individuals tolerated very little additional vertical dimension beyond the minimum tray thickness. Importantly, we did not intend to select a particular horizontal or vertical position at the outset. The clinician simply followed the 300i/SS and when the patient did not respond, the cephalometric radiograph was consulted to evaluate the vertical dimension and impact on airway space.

Fleury et al. used a Nonin 8500M oximeter, which records SpO_2_ to the whole number. Our protocol is novel due to the use of the Minolti 300i/SatScreen software. The 300i records to the 0.1%, giving more detailed SpO_2_ data. An algorithm can then be applied to the data wave to screen out physiologically impossible data, mostly as a result of movement artifacts. Cheyne-Stokes and hypoventilation patterns can also be detected. The extent of information is what allowed for more precise titration than has been previously described in the dental/medical literature, allowing for the discovery of the previously unreported anatomic norms.

The initial starting horizontal protrusion described by Fleury et al. is greater than our final point of protrusion with titration utilizing the 300i/SS. One of the primary morbidities with MAD therapy is the untoward movement of teeth. Literature shows that for every 1 mm of horizontal protrusion, 120.6 g of force are applied to the dentition (50). Three millimeters starting protrusion is an arbitrary starting point, as is 70% of maximum protrusion. The goal of the titration is to put as little force on the teeth as is possible. Fleury's mean mandibular advancement was 11.6 ± 2.6 mm; the titration process presented in this paper had a mean protrusion of 6.2 ± 1.3 mm. The 7.5 mm is the outer edge of the treatment range and relates to the anatomic norms spoken of earlier. The underlying message is that the appliance described here is far less likely to move teeth than one with more protrusion. Twice the protrusion, as reported by Fleury et al., is simply twice the force and therefore, presents a high probability of tooth movement.

As for more recent remote-controlled titration methods during PSG, the protrusion is excessive by the clinical standards represented. There is tremendous bulk of the oral device used to establish the titration position, as opposed to minimal thickness of material as described by this technique.

The goal of the study is to create a MAD that is simple to adjust and forgoes a cumbersome titration process. This would make the appliance available to a much greater segment of the population and lower the cost of setting up and delivering such a device.

Future research directions include long-term efficacy studies for oral appliance patients who underwent the current titration method and concordance of 300i/SS with long-term efficacy of the MAD. Crossover studies using this Herbst-style MAD and CPAP are warranted. Given that 76.2% of the cohort consisted of individuals who did not tolerate CPAP, the present study clearly reflected a group with a higher degree of difficulty. Thus, another potential future study should evaluate untreated OSA patients at diagnosis who would be assigned to MAD as their first line of treatment, to mitigate the potential confounder effect of a CPAP failure on their outcomes. Exploring the relationship between heart rate variability measures on the SatScreen reports and the presence of temporomandibular joint pain, as well as correlation of resolution of pain with a decrease in heart rate as a result of MAD therapy would additional important topics to evaluate in future studies.

## Conclusion

Obstructive sleep apnea (OSA) at any level of severity can be effectively treated with a MAD, even in the presence of obesity or severity > REI of 30/hour. Age and horizontal protrusion emerging as the only two predictive factors associated with CR. Attention to the titration process with a combination of carefully standardized lateral cephalometric images and 300i/SS testing are important to guide the adjustment of MAD and optimize CR rates. The RDI indicator on the SatScreen report indicated even milder desaturation events as the MAD titration changed events frequency and severity, and as such resulted in no further MAD changes being necessary after the second, clearing PSG or HST. The Minolta 300i with SatScreen software is a simple and low-cost method that allows multiple nights of home study and the SatScreen RDI indicator correlates with the final device efficacy. Since the 300i/SS exhibits a closely related final efficacy study to that of the PSG/HST, the treatment results would typically be similar between these two measures. Using the 300i/SS as a clearing study for the MAD could save costs and time while potentially allowing for similar quality of care. Mandibular advancement is an important option when positive airway pressure therapy is either unsuccessful/rejected or if the patient would prefer an MAD initially. The use of a MAD for resolving symptomatic joint pain/TMD could also be of benefit to the patient, and based on the fact that 89 of 101 patients self-report were using their MAD nightly after one-year post-clearing, the adoption and adherence rates seem to be favorable, even if they will need to be corroborated by more objective studies.

## Ethics Statement

The study was carried out in accordance with recommendations of the Institutional Review Board, Feinberg School of Medicine: Northwestern University (Chicago, IL, USA). The protocol was approved by Panel C of the Institutional Review Board at Northwestern. All subjects gave written informed consent in accordance with the Declaration of Helsinki prior to initiating treatment.

## Author's Note

No payment was received from a 3rd party for any aspect of the submitted work. No financial relationships exist with entities that could be perceived to influence the submitted work. The research appliance is generic-no patents or copyrights exist. No other relationships or activities exist that readers could perceive as having influenced the study. No existing relationships exist with the editor; no Frontiers Research Topic is being hosted with the editor. No collaboration exists with any of the authors within the past 2 years. No collaboration exists now or in the past 5 years with any of the authors as an advisor. No collaborations with any of the authors exist as a student in the past 5 years. No one is affiliated with the same institution as the editor; no one is a current member of a committee or department that coincides with an affiliation with the editor. Three of the authors are research employees. No financial interests or business relations exist with any organization involved in the research or manuscript preparation. No financial interest or competing interests exist in the content of the manuscript that might impact the ability to perform an objective review.

## Author Contributions

JM, HA, and DG: conception and design. JM, JB, and CT: acquisition of data. JM, HA, MH, JB, DS, and DG: analysis and interpretation of data and drafting article and critical revision. JM, HA, MH, JB, CT, DS, and DG: final approval for submission.

### Conflict of Interest Statement

The authors declare that the research was conducted in the absence of any commercial or financial relationships that could be construed as a potential conflict of interest.

## Abbreviations

OSA: Obstructive sleep apnea
MAD: Mandibular advancement device
AHI: Apnea-hypopnea index
PSG: Polysomnogram
CPAP: Continuous positive airway pressure
AASM: American academy of sleep medicine
HST: Home sleep test
300i/SS: Minolta 300i/SatScreen
ESS: Epworth sleepiness scale
EDS: Excessive daytime sleepiness
GER: Gastroesophageal reflux
HRPO: High-resolution pulse oximetry
RDI: Respiratory disturbance index
RERA: Respiratory effort related arousal
PAS: Posterior airway space
REI: Respiratory event index
MT: Monitoring time
CR: Complete responder
PR: Partial responder
NR: Non-responder
BMI: Body mass index
TMD: Temporomandibular disorder.
